# Glycopyrrolate in toxic exposure to ammonia gas

**DOI:** 10.4103/0974-2700.76830

**Published:** 2011

**Authors:** A Bhalla, S Mahi, N Sharma, S Singh

**Affiliations:** Department of Internal Medicine, Post Graduate Institute of Medical Education and Research, Chandigarh – 160 012, India

**Keywords:** Ammonia, anti-cholinergic, glycopyrrolate, hoarseness of voice, inhalational injury, toxic gas inhalation, toxic gas, vocal cord injury

## Abstract

Ammonia (NH_3_) is a highly water-soluble, colorless, irritant gas with a unique pungent odor. Liquid ammonia stored under high pressure is still widely used for refrigeration in cold stores used for storing grains. Severe toxicity may occur following accidental exposure. We report an interesting case of accidental exposure to ammonia treated with glycopyrrolate along with other supportive measures.

## INTRODUCTION

Ammonia (NH_3_) is a highly water-soluble, colorless, irritant gas with a unique pungent odor. Liquid ammonia stored under high pressure is still widely used for refrigeration in cold stores used for storing grains in developing countries. Ammonia is also used in the production of explosives, pharmaceuticals, pesticides, textiles, leather, flame-retardants, plastics, pulp and paper, rubber, petroleum products and cyanide. It is a major component of many common household cleaning and bleaching products (e.g., glass cleaners, toilet bowel cleaners, metal polishes, floor strippers, wax removers, smelling salts).[1–3] Although the pungent odor usually acts as a warning of exposure and may prevent further contamination, severe exposure can nevertheless occur.

We report an interesting case of accidental exposure to ammonia treated with glycopyrrolate along with other supportive measures.

## CASE REPORT

A 45-year-old male, a chronic smoker with past history of acute exacerbations of COPD was brought to the emergency department of our institute following an accidental exposure to ammonia gas at a cold storage when the pipe burst. He escaped the place of accident but started complaining of severe burning in the eyes and throat as well as progressive shortness of breath. He was brought to the emergency by his colleagues and the history was obtained from them. On arrival in the ER, he was breathless. His eyes were congested and he was finding it difficult to talk. On examination, he had tachycardia (pulse rate 110/min) and tachypnea (respiratory rate 24/min) but the blood pressure was 130/90 mmHg. The examination of the oral cavity revealed copious pink frothy secretions. Chest examination revealed bilateral polyphonic wheeze. He was drowsy but was able to respond to commands and the oxygen saturation was 85% at room air.

Decontamination was done by removing all his clothes and irrigating the eyes with normal saline using nasal prongs. Both methylprednisolone (125 mg intravenously) and nebulized salbutamol were administered, the latter being difficult to perform due to copious secretions in the oral cavity. The patient was shifted to medical intensive care unit for further management. At this point intubation was contemplated as vigorous suction failed to get rid of the secretions. It was decided by the treating team to first administer 0.2 mg of glycopyrrolate injection to reduce the secretion and try intubation if this failed.

Within minutes the secretions reduced significantly. The patient was nebulized using beta 2 agonists again. He improved significantly and oxygen saturation of 95% was achieved on a FiO2 of 0.3 delivered by ventimask. He was started on prophylactic antibiotics and steroids were continued along with beta 2 agonists for another 48 hours. No further dose of glycopyrrolate was needed. Indirect laryngoscopy was performed to evaluate hoarseness of voice and it revealed an edematous vocal cord.

Topical cycloplegics were used to reduce eye discomfort. Within next 48 hours the patient improved significantly. The chest X-ray and the high resolution CT scan of chest did not reveal any significant alveolar damage. A pulmonary function test revealed mild obstruction. The patient was discharged after a 72 hour stay in the intensive care unit.

## DISCUSSION

Injury from ammonia most commonly is caused by inhalation; it also may follow ingestion or direct contact with eyes or skin.[1] Ammonia gas causes damage when anhydrous ammonia (liquid or gas) reacts with tissue water to form the strongly alkaline solution of ammonium hydroxide. This reaction is exothermic and capable of causing significant thermal injury. It may cause severe alkaline chemical burns to skin, eyes, and especially the respiratory system.[1,2]

Because of its high water solubility, ammonia has a tendency to be absorbed by the water-rich mucosa of the upper respiratory tract. Unlike most highly water-soluble irritant gases that tend to affect exclusively the upper respiratory tract, ammonia can damage respiratory tract both proximally and distally.[1,4] Formation of ammonium hydroxide causes tissues to breakdown, liberating copious amounts of water, and thus perpetuating the conversion of ammonia to ammonium hydroxide. This results in continued exposure and irritation of the respiratory tract.

Brief exposures at very high concentrations and continuous exposure at low concentrations can be overwhelming and affect the entire respiratory system.[1,4] In the respiratory tract, it can cause destruction of cilia and the mucosal barrier. Copious secretions, sloughed epithelium, cellular debris, edema, and reactive smooth muscle contraction causes significant airway obstruction especially in a person with underlying reactive airway disease.[4]

Pain (oropharyngeal, retrosternal), dyspnea, hemoptysis, hoarseness of voice, dysphagia, and loss of consciousness are the commonest encountered symptoms and were all present in our patient. In addition our patient presented with respiratory distress, excessive salivation, and bronchorrhea.[1]

Patients with severe and prolonged exposure are at high risk for developing life-threatening laryngeal edema, therefore airway intervention should always be aggressive. Airway patency can be achieved by medical or surgical methods.[1] Intravenous steroids can be tried but one should not delay intubation if respiratory distress is imminent. Indications for intubation include severe respiratory distress (hypoxemia, hypercapnia), stridor, hoarseness, deep facial burns, burns, and depressed mental status. In case of severe laryngeal edema, intubation may be difficult and one should not be hesitant to resort to cricothyrotomy or tracheostomy to open the airway.

Our patient had stridor, dyspnea, excessive secretions, and altered mental state but we could avoid intubation in him by using a combination of steroids and anti-secretory agent like glycopyrrolate. Inhaled beta-2 agonists (salbutamol) in our patient helped in reversing the bronchospasm. Kosenko *et al*,[5] have reported the protective effects of atropine in patients with acute ammonia poisoning but there have been no reports of glycopyrrolate being used in this context We preferred glycopyrrolate over atropine due to its prolonged effect on salivary and mucosal secretions with reduced cardiac side effects.[6]

Glycopyrrolate is a muscarinic inhibitor and is used preoperatively to reduce salivary, tracheobronchial, and pharyngeal secretions. It has been used for its antimuscarinic actions in organophosphate poisoning.[7] Glycopyrrolate, in high dose, can produce xerostomia (dry mouth), blurred vision, photophobia due to mydriasis, and cycloplegia. Xerostomia and cycloplegia might actually benefit the patients with toxic exposure to ammonia gas. The patients with exposure to highly water-soluble gases like formaldehyde, hydrogen chloride, sulfur dioxide can also present with mucosal irritation and secretions; it is possible, although unproven, if glycopyrrolate can benefit these patients.

In our patient, administration of glycopyrrolate and steroids reversed the respiratory symptoms and avoided further morbidity. In case of severe laryngospasm one should not hesitate to intubate or perform surgical procedures to secure the airway.[1]

## CONCLUSION

This case report suggests that anticholinergic agents like glycopyrrolate can be a useful adjunct to the standard treatment in the management of patients having excessive secretions in the upper respiratory tract following accidental exposure to ammonia gas.
